# Data augmentation based malware detection using convolutional neural networks

**DOI:** 10.7717/peerj-cs.346

**Published:** 2021-01-22

**Authors:** Ferhat Ozgur Catak, Javed Ahmed, Kevser Sahinbas, Zahid Hussain Khand

**Affiliations:** 1 Simula Research Laboratory, Fornebu, Norway; 2 Center of Excellence for Robotics, Artificial Intelligence and Blockchain (CRAIB), Department of Computer Science, Sukkur IBA University, Sukkur, Pakistan; 3 Department of Management Information System, Istanbul Medipol University, Istanbul, Turkey; 4 Department of Computer Science, Sukkur IBA University, Sukkur, Pakistan

**Keywords:** Convolutional neural networks, Cybersecurity, Image augmentation, Malware analysis

## Abstract

Due to advancements in malware competencies, cyber-attacks have been broadly observed in the digital world. Cyber-attacks can hit an organization hard by causing several damages such as data breach, financial loss, and reputation loss. Some of the most prominent examples of ransomware attacks in history are WannaCry and Petya, which impacted companies’ finances throughout the globe. Both WannaCry and Petya caused operational processes inoperable by targeting critical infrastructure. It is quite impossible for anti-virus applications using traditional signature-based methods to detect this type of malware because they have different characteristics on each contaminated computer. The most important feature of this type of malware is that they change their contents using their mutation engines to create another hash representation of the executable file as they propagate from one computer to another. To overcome this method that attackers use to camouflage malware, we have created three-channel image files of malicious software. Attackers make different variants of the same software because they modify the contents of the malware. In the solution to this problem, we created variants of the images by applying data augmentation methods. This article aims to provide an image augmentation enhanced deep convolutional neural network (CNN) models for detecting malware families in a metamorphic malware environment. The main contributions of the article consist of three components, including image generation from malware samples, image augmentation, and the last one is classifying the malware families by using a CNN model. In the first component, the collected malware samples are converted into binary file to 3-channel images using the windowing technique. The second component of the system create the augmented version of the images, and the last part builds a classification model. This study uses five different deep CNN model for malware family detection. The results obtained by the classifier demonstrate accuracy up to 98%, which is quite satisfactory.

## Introduction

Recently our usage of technical gadgets has increased due to the aggressive invasion of technology in our daily life. The frequency of use for many devices has increased many folds, including mobile phones, laptops, webcams, etc. Motivated by market demand, the manufacturers have started to produce devices with attractive features ignoring the security weakness caused by offering such features. Due to the fierce competition among the manufacturers and rapid product development, many products are released to the market with security weaknesses. This offers many opportunities for malicious software developers. Malicious software, commonly known as malware, is intentionally designed to damage computer systems and exploit security weaknesses. Malware is designed for a specific target, often attempting to camouflage itself in another way, with intentions such as file encryption, ransom, preventing a system from working, gaining unauthorized access to a network, data theft, or sabotage. Malware targets various platforms such as servers, personal computers, mobile phones, and cameras to disrupt the system’s normal function. Malware development has become a serious activity lately, and in the only first quarter of 2020, around 1046.10 million new malware has been found (https://www.av-test.org/en/statistics/malware/).

Malware has acquired advanced competencies and diversity in features, which significantly raises the importance of cybersecurity. Cybersecurity activities in various organizations have increased (Shamshirband et al., 2020; Shamshirband & Chronopoulos, 2019) due to its vital importance to the aforementioned problem. One of the essential cybersecurity activities is malware analysis. In order to be effectively protected from malware, the first thing to do is to recognize the malicious software and analyze their behavior well. In this respect, the critical point is to identify malicious software and classify them successfully. A family of malicious software also represents the malicious behavior to which it belongs. As a result, the countermeasures to be taken against these behaviors may vary according to malicious software families. Several consecutive operations are generally performed within malware analysis. This task is mainly done using static and dynamic analysis methods, including the strings command to get the malicious IP addresses, entropy value if the suspicious executable file, executing the file in an isolated environment to record its behaviour.

**Figure 1:**
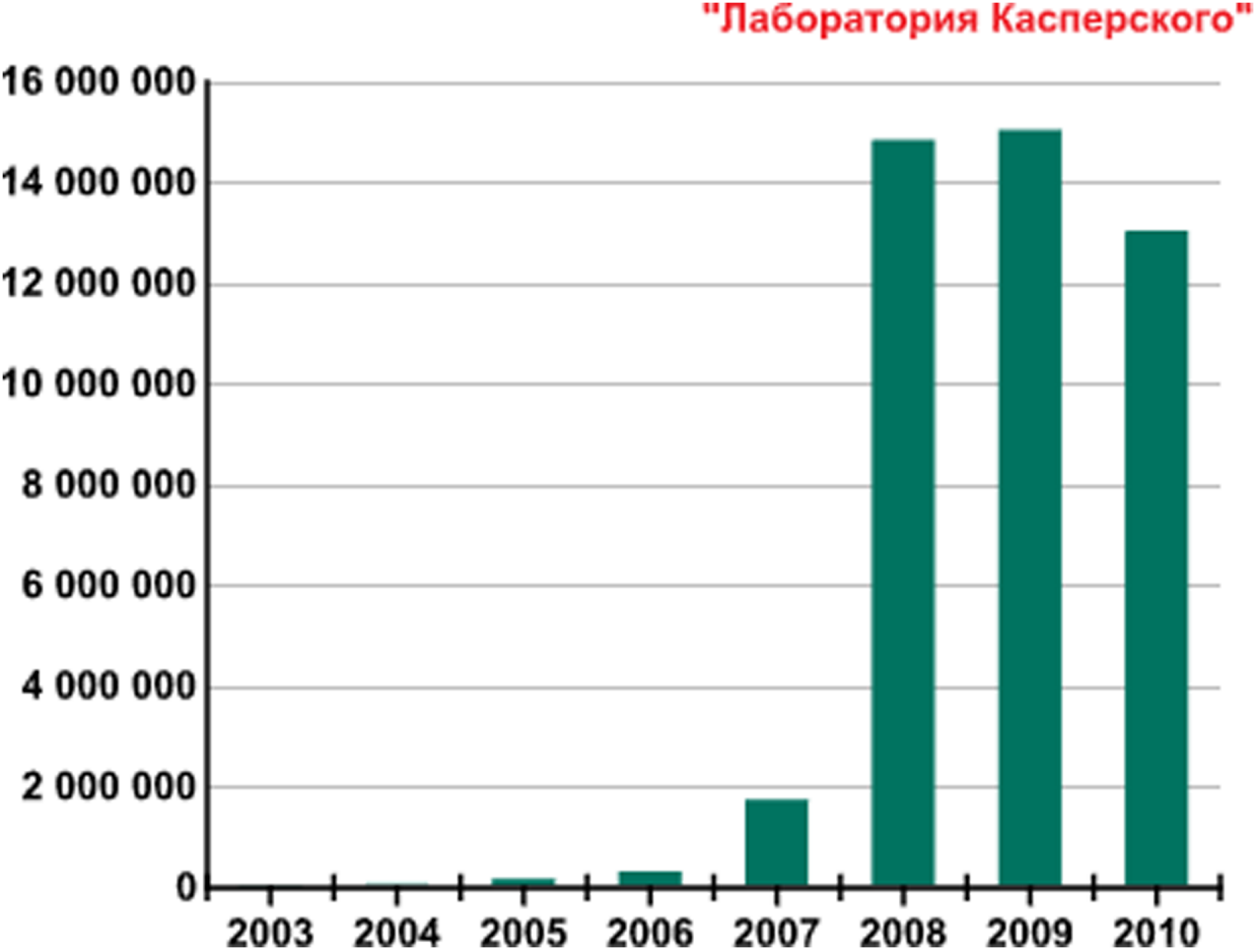
Number of new malicious programs identified per year from 2003–2010.

Figure 1 provides the new malicious programs number detected per year from 2003 to 2010. In the period of 2007 and 2008, the number of new threats has increased significantly due to an increase in the power of antivirus centers processing threats and the evolution in file-infecting technologies. In 2009 almost the same number of new malicious programs was detected as approximately 15 million (https://securelist.com/kaspersky-security-bulletin-2009-malware-evolution-2009/36283/). In 2010, malware evolution has been almost identical to the previous one (https://securelist.com/kaspersky-security-bulletin-malware-evolution-2010/36343/).

**Figure 2:**
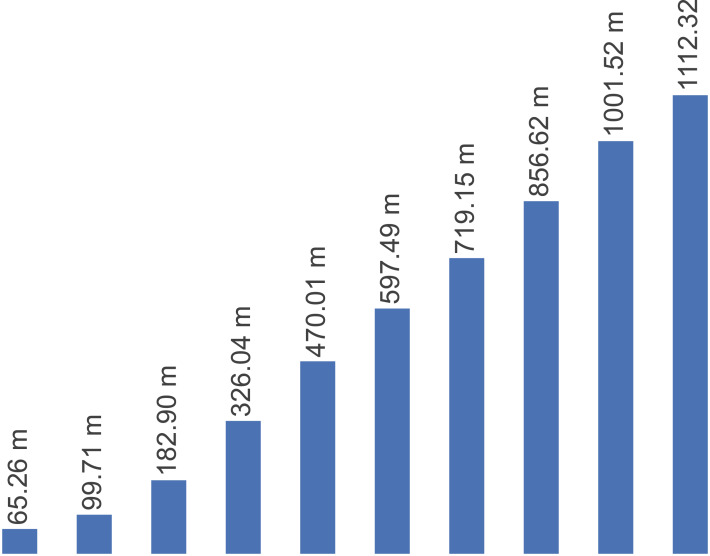
Number of new malicious programs identified per year from 2011–2020.

Figure 2 presents the number of new malware identified per year from 2011 to 2020. It is observed a noticeable increase in the number of new malicious programs year by year. Overall, malware activity has increased from 2011 to 2020.

Malware developers, on the other hand, develop a variety of anti-analysis techniques with their broad knowledge of existing analysis methods. Anti-debugging and anti-disassembly techniques are the two methods most commonly used by malware developers. Such methods to bypass analysis are generally used to produce erroneous results by the disassembler and debugger tools. In anti-debugging methods, malware developers often manipulate pointer address parameters used by jump op-code such as jz, jnz, jze. Anti-debugging techniques are used by the developers to ensure that malware samples do not run under a debugger, and in that case, change the execution flow accordingly. In most cases, the reverse engineering process will be slow down by the anti-debugging technique. The automated malware detection systems used these days do not yield very successful results due to the aforementioned reasons. Proper malware labeling is a challenging issue in this domain. An anti-virus application can detect malware as a trojan, whereas the same malware is labeled as a worm by another anti-virus application. It has become even more complicated with the advent of sophisticated malware.

With the development of machine learning, it has been observed that these techniques are being used in the field of malware analysis. To use API calls as the feature vector is one of the first usages of machine learning algorithms for malicious software analysis (Mira, 2019). N-grams are other commonly used methods for the quantification of API calls. The main reasons for using n-grams are to reduce computation-complexity of the model, to create a simple term-frequency × inverse-document-frequency (TF-IDF) matrix, and to use traditional algorithms such as random forests, decision tree, and support vector machine (SVM). Although such an approach has produced high classification performance results, they remain inadequate for the current malware infection methods. Malware analysts need sandbox applications to create API call datasets because a sandbox provides an isolated virtual machine (VM) with a secure and close network environment. The behaviour of malicious software runing in the VM are recorded.

However, malware developers use anti-VM and anti-sandbox methods that integrate various virtual machine detection code snippets into their malicious code blocks. If the malicious software gets the impression of executing on a virtual machine or sandbox environment, then it changes its behaviour to complicate the analysis. The most widely used anti-VM and anti-sandbox methods are “Checking CPUID Instruction”, “VMWare Magic Number”, “Checking for Known Mac Addresses”, “Checking for Processes Indicating a VM”, “Checking for Existence of Files Indicating a VM” and “Checking for Running Services”. Although malware changes its behaviour and blocks dynamic analysis, some machine learning methods can be used to obtain malware families depending on the malware code. Currently, the approach used for malware analysis is based on creating a grayscale image from malware code and then using classification algorithms.

We created classification models by extracting only the behaviour of malware samples in our previous works (Catak & Yazı, 2019; Yazı, Catak & Gul, 2019; Catak et al., 2020). We executed all the malware samples in the Cuckoo sandbox environment. whereas, in this study, harmful software did not operate in an isolated sandbox environment. This research’s main contribution is to develop a data augmentation enhanced malware family classification model that exploits augmentation for malware variants and takes advantage of a convolutional neural network (CNN) to improve image classification. Herein, we demonstrate that the data augmentation-based 3-channel image classification can significantly influence malware family classification performance. Malware developers use different methods to camouflage the malicious behaviour of malware while executing. There is no real execution phase in an operating system in our approach. Another technique that malware developers apply is to put various modifications (such as noise) to the content when they propagate from one computer to another. We used data augmentation methods to solve this camouflage technique to our malware image samples to detect their variants.

The rest of the article is organized as follows: “Related Work” briefly describes the related work. In “System Model”, we present the system model and consists of two subsections. The first subsection presents the image conversion, and the second subsection presents the data augmentation. “Proposed Approach” provides fine-grained details of the proposed approach and presents the malware classification algorithm. “Experiments” provides an extensive analysis of results. Finally, in “Conclusion and Future Work”, we conclude the article and present some future research directions.

## Related work

Malware analysis field has gained considerable attention from research community with rapid development of various techniques for malware detection. There is huge research literature in this area. Since the proposed work is related to image-based analysis using deep learning techniques, the relevant research literature regarding image processing techniques for malware detection are briefly discussed in this section. One of the early studies conducted on malware images was done by Nataraj et al. (2011). The authors proposed an image texture analysis-based technique for visualization and classification of different families of malware. This approach converts malware binaries into grayscale images. Malware are classified using K-nearest neighbor technique with Euclidean method. However, the system requires pre-processing of filtering to extract the image texture as features for classification.

On the other hand, to extract the image texture as features for classification, the system requires pre-processing of filtering. Kancherla & Mukkamala (2013) proposed a low-level texture feature extraction technique for malware analysis parallel to Nataraj’s technique. The authors converted malware binaries into images and then extracted discrete wavelets transform based texture features for classification. Makandar & Patrot (2017) identify new malware and their variants to extract wavelet transforms-based texture features, and then supply to feed forward artificial neural network for applying classification. Kosmidis & Kalloniatis (2017) described a two-step malware variant detection and classification method. In the first step, binary texture analysis applied through GIST. In the second step, these texture features classified by using machine-learning techniques such as classification and clustering to identify malware. Although the works mentioned above Nataraj et al., 2011; Kancherla & Mukkamala, 2013; Makandar & Patrot, 2017; Kosmidis & Kalloniatis (2017) are helpful to detect and classify new malware and their variants, they still have some limitations. For instance, on the one hand, global texture features lose local information needed for classification. On the another hand, they have significant computation overheads to process a vast amount of malware.

According to Zhang et al. (2016), the malware classification problem can be converted into an image classification problem. Their study provides to disassembles executable files into opcode sequences and then convert opcode into images for identifying whether the source file is benign or malware by using CNN. Yue (2017) presents multifamily malware classification approach by applying CNN. However, the performance is degraded due to the imbalance of malware families. The author proposes softmax loss function to mitigate this issue. This approach is reactive in nature to deal with scenarios where class imbalance is assumed.

The other work by Ni, Qian & Zhang (2018) propose a method for malware classification by applying deep learning techniques. Their algorithm uses SimHash and CNN techniques for malware classification. The algorithm converts the malware codes that is disassembled into grayscale images used SimHash algorithm and after that uses CNN to identify their family. The performance improvement is ensured by using some methods such as bilinear interpolation, multi-hash and major block selection during the process. Cui et al. (2018) propose a method that applies CNN with the Bat algorithm together in order to robust the accuracy of the model. Their implemented method converts the malicious code into grayscale images. The method’s images are classified by using a CNN and Bat algorithm is used to address the issue of data imbalance among different malware families. The main limitation of this approach is that they used one evaluation criterion to test the model. The other work by Nisa et al. (2020) suggest a new approach using malware images with rotate, flip and scale base image augmentation techniques.

Two stage deep learning neural network is used by Tobiyama et al. (2016) for infection detection. Initially, the authors generated an image via the extracted behavioral features from the trained recurrent neural network. Later, to classify the feature images, they used CNN. An approach to derive more significant byte sequence in a malware was proposed by Yakura et al. (2018). The authors used CNN with attention mechanism to achieve this for the images converted from binaries. MalNet method for malware detection was proposed by Yan, Qi & Rao (2018). The method automatically learns essential features from the raw data. The method generates grayscale images from opcode sequences. Later, CNN and LSTM are used to learn important features from the grayscale images. Fu et al. (2018) proposed an approach to visualize malware as an RGB-colored image. Malware classification is performed by merging global and local features using random forest, K-nearest neighbor, and support vector machine. The approach realizes fine-grained malware classification with low computational cost by utilizing the combination of global and local features. Liu et al. (2019) proposed a malware classification framework based on a bag-of-visual-words (BoVW) model to obtain robust feature descriptors of malware images. The model demonstrates better classification accuracy even for more challenging datasets. The major limitation of this approach is higher computational cost.

Chen et al. (2019) conducted an extensive study on the vulnerabilities of the CNN-based malware detectors. The authors proposed two methods to attack recently developed malware detectors. One of these methods achieve attack success rate over 99% which strongly demonstrates the vulnerability of CNN-based malware detectors. The authors also conducted experiments with pre-detection mechanism to reject adversarial examples and shown its effectiveness in improving the safety and efficiency of malware detectors. Venkatraman, Alazab & Vinayakumar (2019) used similarity mining and deep learning architecture to identify and classify obfuscated malware accurately. The authors used eight different similarity measures to generate similarity matrices and to identify malware family by adopting images of distance scores. The advantage of this approach is that it requires less computational cost as compared to classical machine learning based methods. Dai et al. (2018) proposed a malware detection method using hardware features due to inherent deficiencies in software methods. The approach dumps the malware memory of runtime to binary files, then grayscale image is extracted from the binary files. A fixed size images are generated from the grayscale image and histogram of gradient is used to extract image features. Finally, malware classification is done using the popular classifier algorithms. One of the limitations for this approach is that it cannot provide against fileless malware. Gibert et al. (2019) propose a file agnostic deep learning approach for malware classification. The malicious software are grouped into families based on a set of discriminant patterns extracted from their visualization as images. Yoo, Kim & Kang (2020) propose multiclass CNN model to classify exploit kits. On of the root of malware contamination are exploit kits. This type of attack has rapidly increased and detection rate is quite low. The authors proposed limited grayscale, size-based hybrid model and recursive image update method to enhance classification accuracy.

Traditional machine learning methods are applied in most of the existing state of the art. Our study uses a deep learning method and differs from most other studies examined in this section. Deep learning methods are not algorithmically new and easy to implement. They can be trained with high-performance computations on systems such as GPUs. Today, they have become prevalent in the field of machine learning. Some of the studies examined also used deep learning methods, but our approach differs from these studies because we used five different deep CNN models for malware family classification. It is evident from the results that 3-channel image classification can significantly influence malware family detection’s performance. The main contribution that makes this study stand out regarding the existing state of art examined in this section is applying data augmentation enhanced malware family classification model. This model exploits augmentation for variants of malware clones and take advantage of CNN to improve image classification.

## System model

The system architecture of the proposed model is composed into three different components. The first component is image conversion of malware samples using decimal representation and entropy values of each byte. The second component is image augmentation component. The last one is CNN based malware family classification.

### Image conversion

**Figure 3:**
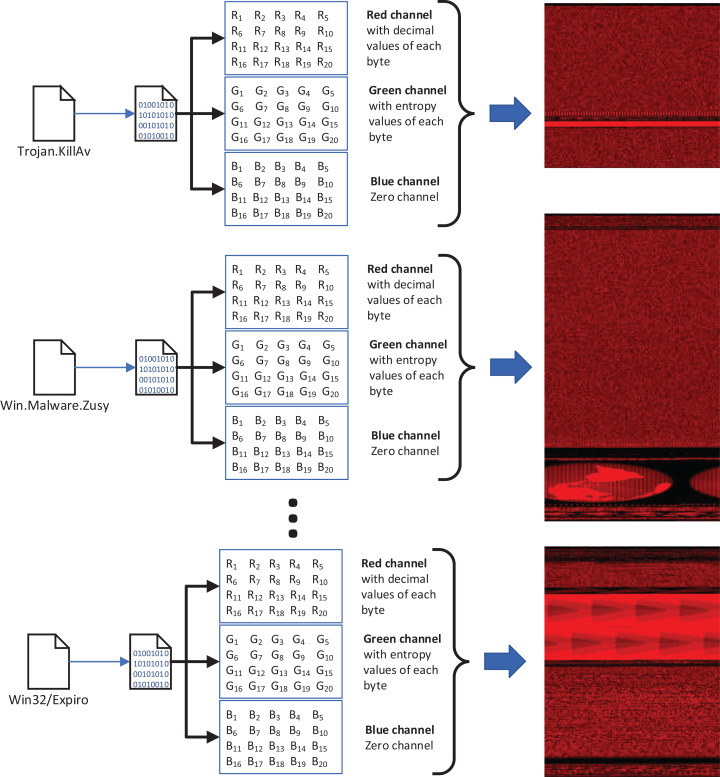
The architecture of the proposed 3-channel image representation of malware samples. Given input malware samples, RGB representations are computed by applying as explained in “Basic Idea”.

We used our publicly available malware dataset for this approach (https://github.com/ocatak/malware_api_class. Malware samples collected from Github platform, in the first step, are labeled using *ClamAV* open source command-line antivirus software. The model architecture is illustrated in Fig. 3. Every malware sample is split into their bytes. In the second step, each byte is converted from bit representations to decimal representation for the red channel. For instance, the byte representation with 10010110 is converted to 150 as the decimal representation. In the third step, we calculated the entropy value of the byte representations. As an example of the same byte value of 10010110, the entropy value is 1.

The input of the first component of the malware detection system is a collection of malware stored in different formats such as portable executable, Word, PDF. These malware are then converted into 3-channels PNG files as shown in Fig. 3.

**Figure 4:**
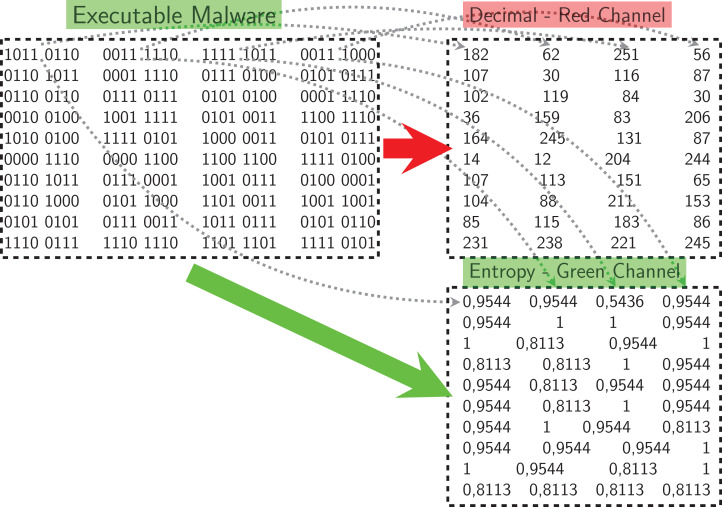
Example process of pixel generation from the opcode.

Figure 4 shows an example pixel generation process. Each byte value of the executable file is converted to its decimal representation for the red-channel, and the corresponding entropy value for the blue-channel.

### Data augmentation

The key problem with malware detection model is data diversity. There are many alternative methods are available for solving these problems. One approach to solve this problem involves the use of data augmentation. Data augmentation can be defined as a strategy to artificially increase the variety of input instances for training phase, without really collecting new instances.

Additive noise is the most used technique for data augmentation to build reliable machine learning models. Gaussian, Laplacian and Poisson noises are the most used techniques to enhance the input dataset. Laplacian noise is derived eventually from white (Gaussian) noise (Hida & Si, 2008). They are the most used additive noise techniques to improve and enhance the image datasets (Harmsen & Pearlman, 2003; Holmstrom & Koistinen, 1992).

#### Additive Gaussian

Additive Gaussian noise is a fundamental noise model used in information theory to simulate the impact of many random methods that happen in nature (Selesnick, 2008). The Additive Gaussian noise flow is represented by a series of outputs *Y*_i_ at a discrete-time event index *i*. *Y*_i_ is the sum of the input *X*_i_ and noise, *Z*_i_, where *Z*_i_ is independent and identically distributed and picked from a zero-mean normal distribution, including variance *N*. The *Z*_i_ are further assumed to not be correlated with the *X*_i_.

(1) $$\matrix{ {{Z_{\rm i}}} \hfill \,\,\,{{\rm \sim {\mathcal N}}(0,N)} \hfill \cr {{Y_{\rm i}}} \hfill { = {X_{\rm i}} + {Z_{\rm i}}} \hfill \cr }$$

#### Additive Poisson

Poisson noise is a kind of noise that can be represented by a Poisson process (Wojtkiewicz et al., 1999). A discrete random variable *X* is said to have a Poisson distribution with parameter λ > 0, if, for *k* = 0, 1, 2, …, the probability mass function of *X* is given by:

(2) $$f(k;\lambda ) = \Pr (X = k) = \displaystyle{{{\lambda ^k}{e^{ - \lambda }}} \over {k!}}$$

where *e* is Euler’s number, and *k*! is the factorial of *k*.

#### Additive Laplace

The Laplace distribution is a continuous probability distribution that sometimes described the double exponential distribution because it can be considered as two exponential distributions with an extra location parameter joined together (Marks et al., 1978).

A random variable has a *Laplace* distribution if its probability density function is

(3) $$\matrix{ {f(x|{\rm \mu} ,b)} \hfill & { = \displaystyle{1 \over {2b}}\exp \left( { - \displaystyle{{|x - {\rm \mu} |} \over b}} \right)} \hfill \cr {} \hfill & { = \displaystyle{1 \over {2b}}\left\{ {\matrix{ {\exp \left( { - \displaystyle{{{\rm \mu} - x} \over b}} \right)} & {{\rm if}\,\,x \lt  {\rm \mu} } \cr {\exp \left( { - \displaystyle{{x - {\rm \mu} } \over b}} \right)} & {{\rm if}\,\,x \ge {\rm \mu} } \cr } } \right.} \hfill \cr }$$

### Malware development

Malware developers try to hide the malicious code snippets they place on legitimate software from malware analysts and antivirus programs using different methods. In addition, malware software developers use codes and frameworks that belong to malware families that perform similar malicious activities, rather than rebuilding malware code fragments. For this reason, when these malware are converted into a executable file (example: PE for Windows) to be suitable for the target platform on which they will be run, they are very similar when binary analysis is performed. The signature-based security components used today are very vulnerable to changes in the code, which reduces their detection capabilities. Developers generally use two different methods to replace the malicious code content when contaminated software infects from one host computer to another computer; *polymorphic* and *metamorphic* malware.

**Figure 5:**
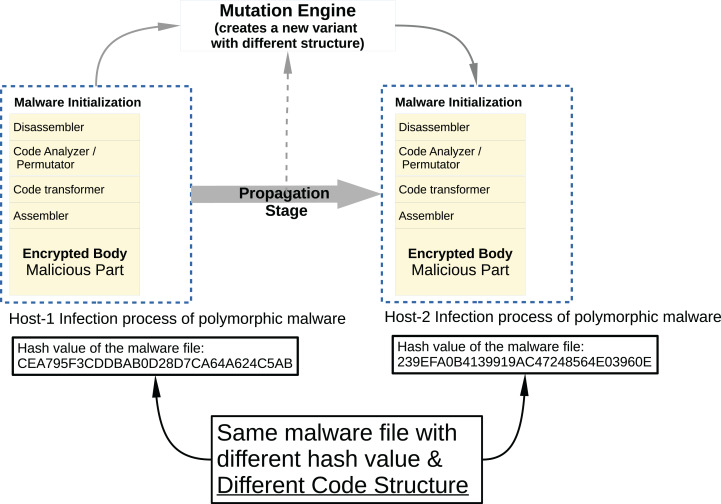
Typical metamorphic malware propagation.

In Metamorphic malware, the situation is a bit more complicated. Although the obfuscation techniques are applied in the same way, this time the code flux is changed. As seen in Fig. 5, a typical metamorphic malware has more components and its structure has become more complex. This time malware has different components such as disassembler, code analyzer/permutator, code transformer, assembler, and malicious payload.

## Proposed approach

This section presents the results of the data augmentation, data enhancement-based CNN malware classification algorithm. The basic idea of Augmented-CNN based malware classification techniques is introduced in “Basic Idea”. The implementation of the porposed technique is described in “Implementation of the Model”.

**Figure 6:**
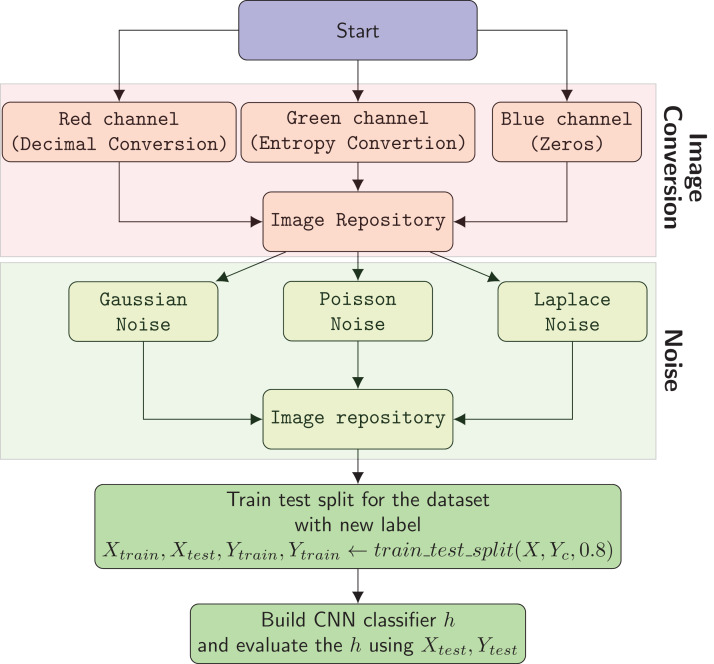
Flow chart of the overall system.

Figure 6 shows the flowchart of the overall method. The process of malware classification includes the following steps in the proposed solution:

- The system creates RGB images using Decimal Conversion, Entropy Conversion and Zeros
- Gaussian, Poisson, and Laplace noises with their combinations are added to images to enhance the input dataset.
- In the third step the system builds a CNN based classification model.

### Basic idea

As previously mentioned in “Malware Development”, malware developers are trying to evade security components using different methods. These methods are usually in the form of adding noise to the executable files’ binary form. One of the areas dealing with noisy data is the image classification task. One of the methods used to overcome this problem and to classify images from different angles in a more reliable way is the image augmentation technique. As part of this study, malware samples have been converted to 3-channel images. The evasion techniques that malware developers have added are reflected in these images as noise. We used image augmentation techniques in this study so that the noise in the images does not affect the classification performance.

**Figure 7:**
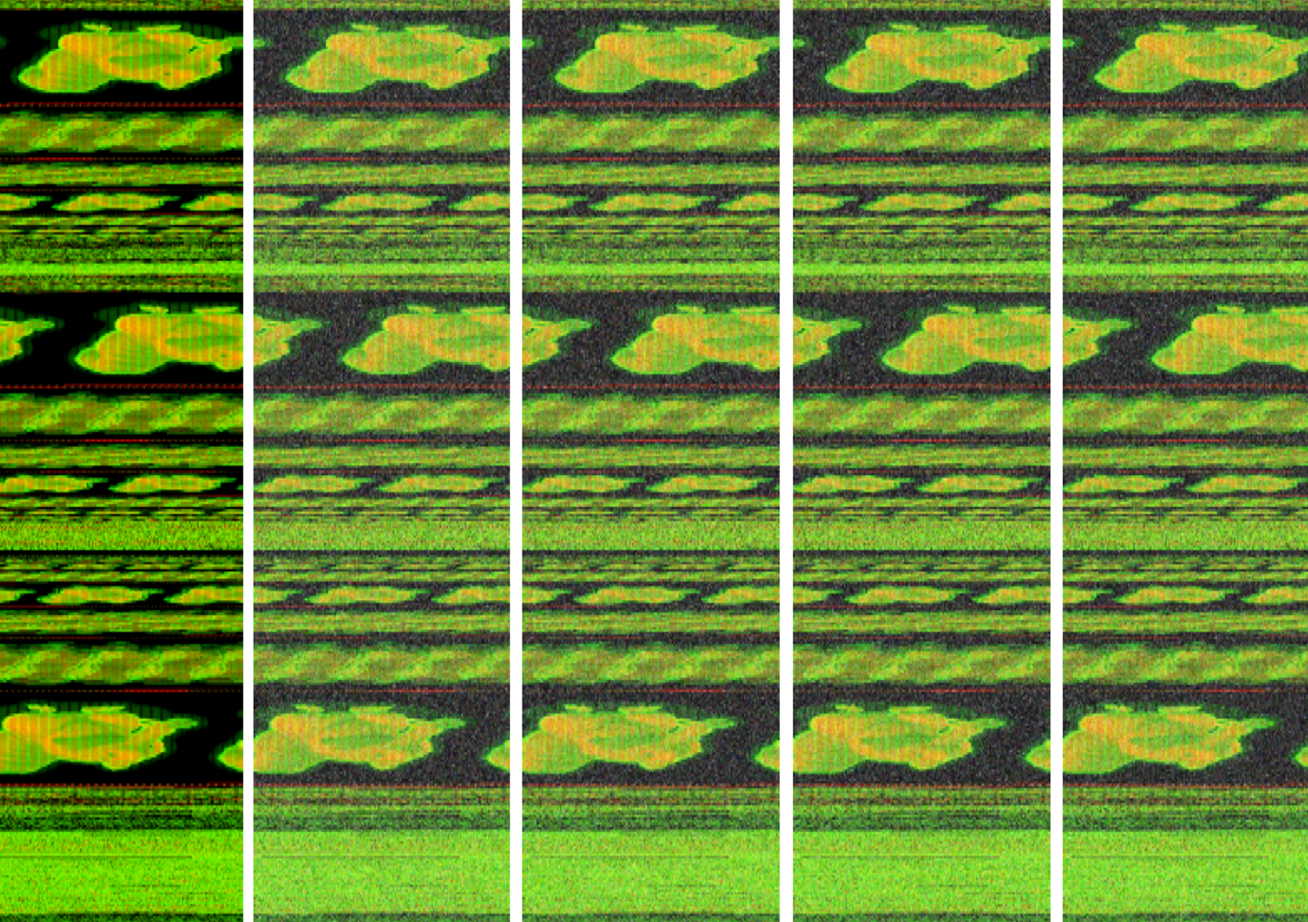
The different additive Laplace noise to Trojan/Win32.VBKrypt.C122300 malware.

We used the *imgaug* Python library for implementation and increased our dataset to 5 times using *AdditiveGaussian*, *AdditiveLaplace* and *AdditivePoisson* noise addition methods. In Fig. 7, new images are created with different laplace noises for *Trojan/Win32.VBKrypt.C122300* malware.

Our main tasks are to enhance data using data augmentation and classify malware samples according their family using malware images based CNN model. Malware images’ basic idea is create multi-channel images using byte streams and entropy values of each 8-bits streams. Table 1 presents notations to evaluate the malware classifier model performance and the commonly used variables is presented for convenience.

**Table 1:** Commonly used variables and notations.

Variables/notations	Description
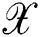	Original input dataset
${\mathcal X}_{aug}$	Augmednted version of input dataset *X*
$f_{aug}^{m}$	Augmentation function *m*
*ε*	Augmentation threshold
Acc	Accuracy of the classifier
*k*	Number of classes
*t*	Number of augmentation functions

### Analysis of the proposed algorithm

The reason behind of this study is the idea that using the law of large numbers theory, we have opportunity to obtain more accurate classifier model (for this work malware classification) by creating new samples that is comparable to original models which are created with original input instances.

In the proposed approach, there is a set of augmentation functions that acts a data creation source for CNN model. The single augmentation function, $f_{aug}^m$, is defined as follows:

(4) $${{\mathcal X}}_{{aug}}^{({m})} = f_{{aug}}^{m}({{\mathcal X}})$$

The each augmented dataset, ${{\mathcal X}}_{aug}^{(m)}$, using each augmentation algorithm, $f_{aug}^{(m)}$, is combined into a single enhanced dataset. The final augmented dataset is defined as follows:

(5) $${{{\mathcal X}}_{{aug}}} = \bigcup\limits_{{i} = 1}^{\rm t} {{\mathcal X}}_{{aug}}^{({i})}$$

where *t* is the number of augmented dataset, ${{\mathcal X}}_{aug}^{(i)}$ is the *i*th augmented dataset.

### Implementation of the model

The pseudocode of transformation of PE executable to multichannel images is shown in Algorithm 1. The each member (*e*^(i)^) of collected Windows executable file set, 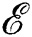, is converted multi-channel images in lines 5-6. For the first channel of the executable, one byte is read and then converted to the decimal representation in line 5. The decimal value is assigned to the first channel of the corresponding pixel, 
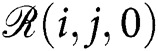
. In the same way, this byte’s entropy value is assigned to the second channel of the corresponding pixel, 
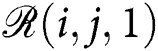
. We used imgaug library which uses 3-channel PNG images as input. On the other hand, we created 2-channel PNG images in this research. Since the imgaug software library requires three channels images, we had to fill the last channel, the Blue channel, with zeros. Accordingly, our algorithm’s both time and space complexity is O(n).

**Algorithm 1:** PE malware to image conversion.

1: **Inputs:** PE executable set 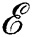 , image width *w*, image height *h*, channel size *c* 2: **for** each *e*^(*i*)^ ∈ 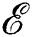 **do** 3: ℛ *zeros*(*w*,*h*, *c*) where ℛ ∈ ℝ^*w*×*h*×*c*^ ⊳ Create a zero filled matrix 4: **for** each byte value *b*^(*j*)^ ∈ *e*^(*i*)^ **do** 5: 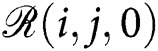 *decimal*(*b*^(*j*)^) ⊳ 1st channel with value ∈ [0,255] 6: 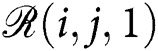 −Σ_*x*∈*b*^(*j*)^_ (*p*(*x*) · log *p*(*x*)) ⊳ 2nd channel with entropy ∈ [0,255] 7: **end for** 8: **end for** 9: **Outputs:** Image dataset 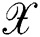

The pseudocode of data-augmentation enhanced CNN malware detection are shown in Algorithm 2. The augmentation procedure is implemented based on random noise assigment of each channel of the training dataset, ${{\mathcal X}}$, with a set of augmentation functions, *F*_aug_.

**Algorithm 2:** Data enhancement.

1: **Inputs:** 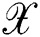 = {{(**x**_*i*_, *y_i_*) | *i* = 1, …, *n*}, **x**_*i*_ ∈ ℝ^*p*^, *y_i_* ∈ {−1,+1}}^*m*^_*i* = 1_, Augmentation function set *F_aug_* 2: Initialize 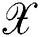 ^(*i*)^_*aug*_ = 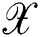 3: **for** each *f*^(*i*)^_*aug*_ ∈ *F_aug_* **do** 4: 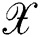 ^(*i*)^_*aug*_ ← *f*^(*i*)^_*aug*_( 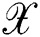 ) 5: 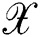 ← 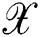 ∪ 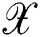 ^(*i*)^_*aug*_ 6: **end for** 7: **Outputs:** Enhanced dataset 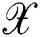

## Experiments

In this section, we use our public malware dataset (https://github.com/ocatak/malware_api_class). that can be accessed publicly. The malware classification model is compared with the original dataset. In “Dataset Detail”, we explain the dataset and parameters that are used in our experiments. The conventional CNN is applied the dataset and we find the classification performance in “Dataset Results with Conventional CNN”. In “Dataset Results with Proposed Method”, we show the emprical results of proposed augmented CNN training algorithm.

### Experimental setup

To our knowledge, there is no public benchmark dataset for malware images approach to make an evaluation comparison. We apply our dataset with different hyper-parameters to indicate the effectiveness and classification performance of the proposed model.

The experiments are done using the Python programming language and machine learning libraries Keras, Tensorflow, and Scikit-learn. We used the Keras library to build CNN networks.

For the experimental setup to generate a model that is able to generalize, we divided the dataset into two partitions: the training set with 80% of the dataset and the testing set with 20% of the dataset. The learning rate for the CNN was 0.01.

### Dataset detail

We trained our classifiers with our public dataset which is summarized in Table 2 with seven different classes including Worm, Downloader, Spyware, Adware, Exploit, Malware and Benign.

**Table 2:** Description of the training dataset used in the experiments.

Malware type	#Inst.
Worm	1,620
Downloader	1,512
Spyware	582
Adware	1,146
Exploit	138
Malware	456
Benign	308
Total	5,762

There are 5,762 malware samples from different classes in this dataset. The Cuckoo Sandbox application, as explained above, is used to obtain the Windows API call sequences of malicious software, and VirusTotal Service is used to detect the classes of malware.

**Figure 8:**
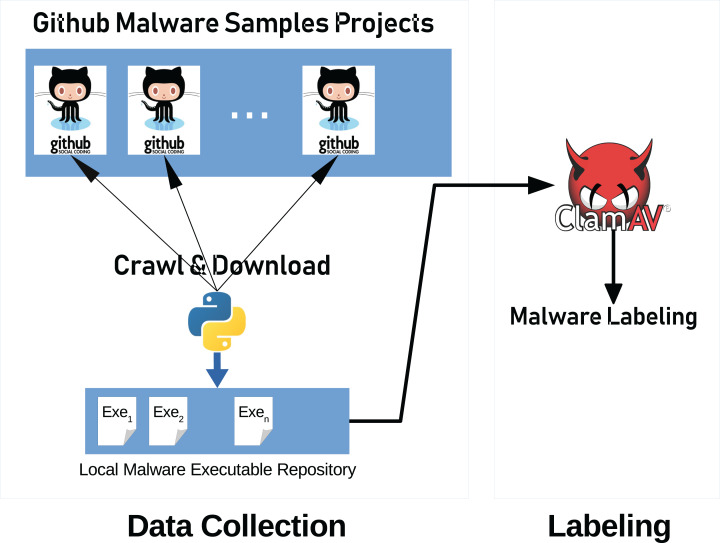
General system architecture. Architecture consists of three parts; data collection, data pre-processing and data classification.

Figure 8 illustrates the system architecture used to collect the data and labeling process. Our system consists of two main parts, data collection, and labeling.

### Evaluation

Although the dataset that is applied in our method is almost balanced, performance evaluation in terms of traditional accuracy not sufficient to obtain an optimal classifier. Besides, we apply four metrics such as the overall prediction accuracy, average recall, average precision Turpin & Scholer (2006) and F1-score, to estimate the classification accuracy that are used as measurement metrics in machine learning common Manning, Raghavan & Schütze (2008) and Makhoul et al. (1999).

Precision is the ratio of predicted positive classes to positive predictions. Precision is estimated in Eq. (6).

(6) $${\rm Precision = \displaystyle{{Correct} \over {Correct + False}}}$$

Recall is the ratio of positive classes to the sum of positive correct estimation and false negative. It can be called Sensitivity. Recall is indicated in Eq. (7).

(7) $${\rm Precision = \displaystyle{{Correct} \over {Correct + Missed}}}$$

First, our proposed evaluation model estimates precision and recall for each and then calculate their mean. In Eqs. (8) and (9), we present average precision and recall.

(8) $${\rm Precision_{avg}} = \displaystyle{1 \over {{n_{\rm classes}}}}\sum\limits_{i = 0}^{{n_{\rm classes}} - 1} \left( {{\rm Prec}{_i} \times {\rm num\_of\_instances}{_i}} \right)$$

(9) $${\rm Recall{_{avg}}} = \displaystyle{1 \over {{n_{\rm classes}}}}\sum\limits_{i = 0}^{{n_{\rm classes}} - 1} \left( {{\rm Recall}{_i} \times {\rm num\_of\_instances}{_i}} \right)$$

The average precision and recall values are calculated using the multiplication of recall and the number of instance in the corresponding class. Precision and Recall are evaluated together in F-measure. It is the harmonic mean of precision and recall. F-measure is provided in Eq. (10).

(10) $${F_1} = 2 {\rm\times \displaystyle{{Pre{c_{{avg}}} \times Recal{l_{avg}}} \over {Pre{c_{avg}} + Recal{l_{avg}}}}}$$

### Dataset results with conventional CNN

**Figure 9:**
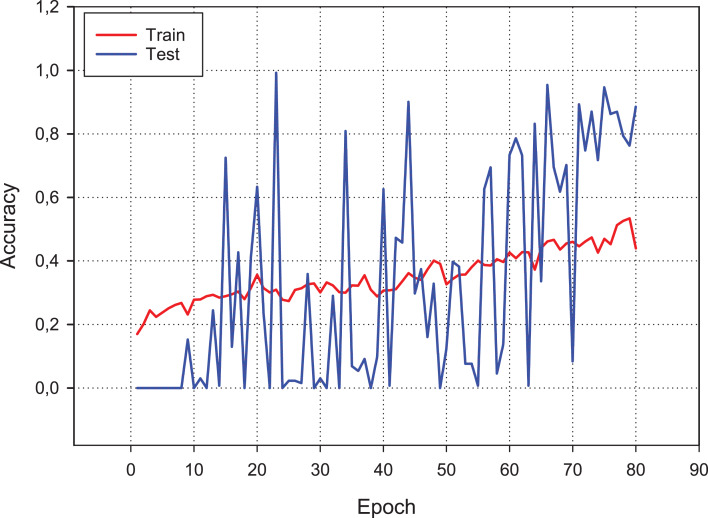
Accuracy changes over learning iterations. As can be seen, although the training dataset shows more stable progress, the test dataset is less stable, although it progresses together.

**Figure 10:**
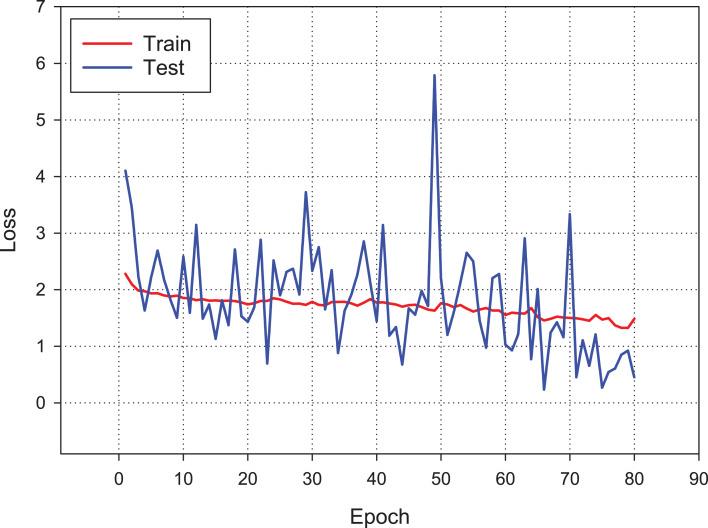
Loss changes over learning iterations. As can be seen, although the training dataset shows more stable progress, the test dataset is less stable, although it progresses together as in Fig. 9.

Figure 9 presents the accuracy performance of the conventional CNN model for our experimental data set. As shown in figure, the model becomes its steady state after 80th epoch. Also, Fig. 10 shows the loss value changes of classification model through epochs.

**Figure 11:**
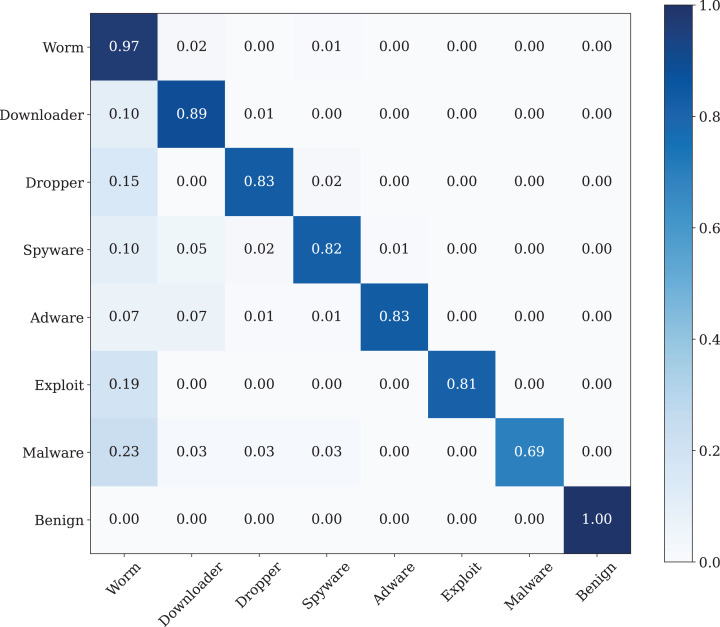
The confusion matrix of the CNN model, which was trained using the original dataset.

A confusion matrix is applied to evaluate the performance of our model. The findings from Fig. 11 show the confusion matrix that was trained by using the original dataset by using CNN model. The findings of the confusion matrix indicate that the classification model performance is not good enough for the malware detection.

The testing classification performance is measured through accuracy, precision, recall and *F*_1_ measure. Table 3 shows the best performance of the conventional CNN method of each malware family.

**Table 3:** Classification report of conventional CNN for each malware class.

	Precision	Recall	*F*_1_
Worm	0.60	0.58	0.59
Downloader	0.82	0.11	0.20
Dropper	0.62	0.05	0.10
Spyware	0.39	0.69	0.50
Adware	0.22	0.72	0.34
Exploit	0.86	0.26	0.40
Malware	0.00	0.00	0.00
Benign	0.77	0.83	0.80

As can be seen from the confusion matrix and classification report, the classification performance of the model obtained with conventional CNN is rather low. According to these results, a standard CNN model with RGB type 3-channel image training dataset is not suitable for malware detection and classification.

### Dataset results with proposed method

**Figure 12:**
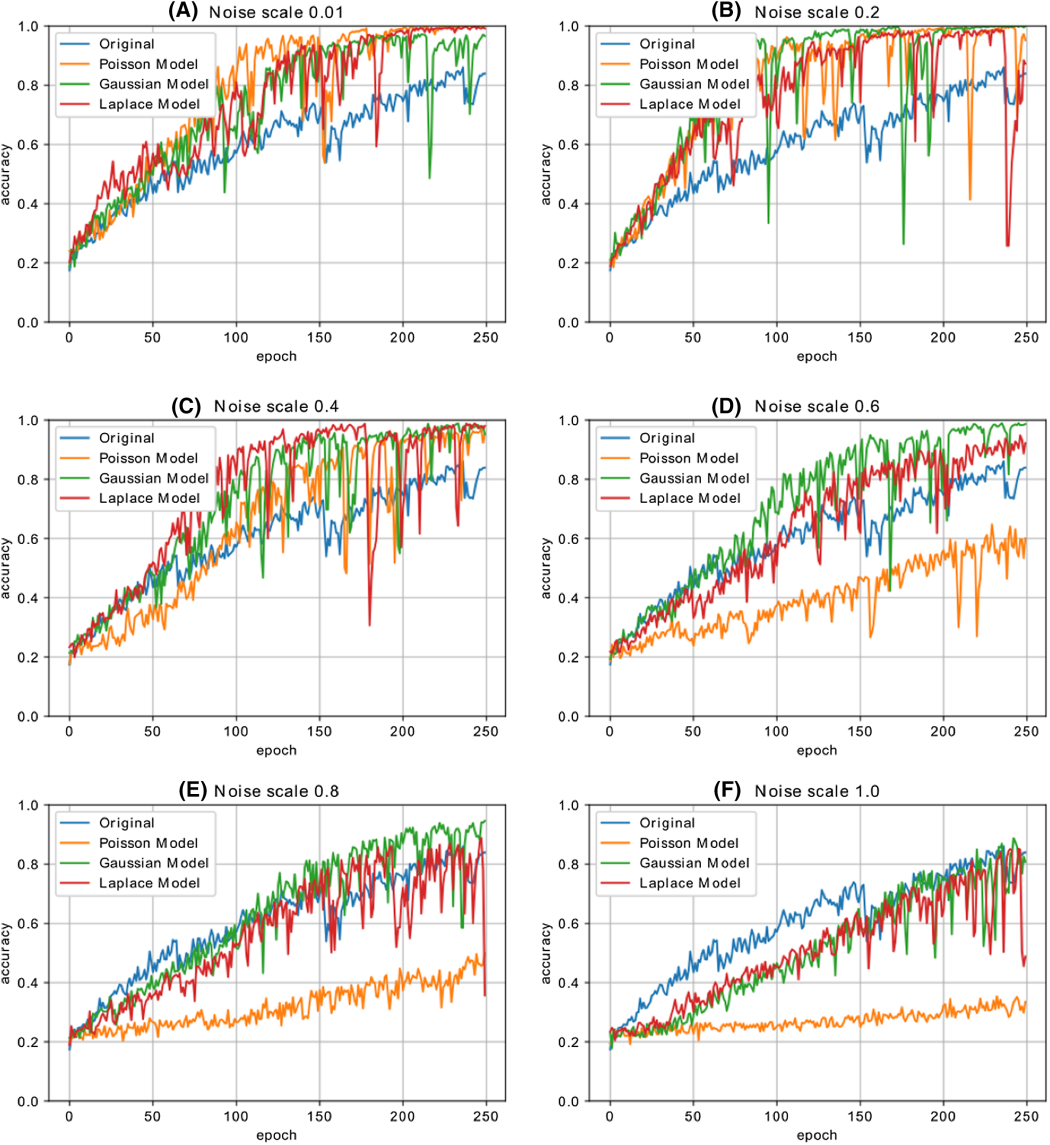
The different noise ratio accuracy results for additive Laplace/Gaussian/Poisson and original CNN model’s accuracy results. Noise scale: (A) 0.01; (B) 0.2; (C) 0.4; (D) 0.6; (E) 0.8 and (F) 1.0.

Figure 12 shows the accuracy change in each iteration of the CNN model, which is trained with the malware dataset containing a different amount of noise. The performance results of four CNN models, whose dataset is enriched by using both Additive Laplace, Additive Gaussian, and Additive Poisson methods, are better than the CNN model’s classification performance that is trained only with the original training data set. When the noise ratio is 0.5, the original CNN model’s classification result is better than the CNN model with the Additive Poisson method. When the noise ratio is increased to 0.8, the classification results of CNN models with Additive Gaussian, Additive Laplace, and Additive Poisson begin to decrease.

**Figure 13:**
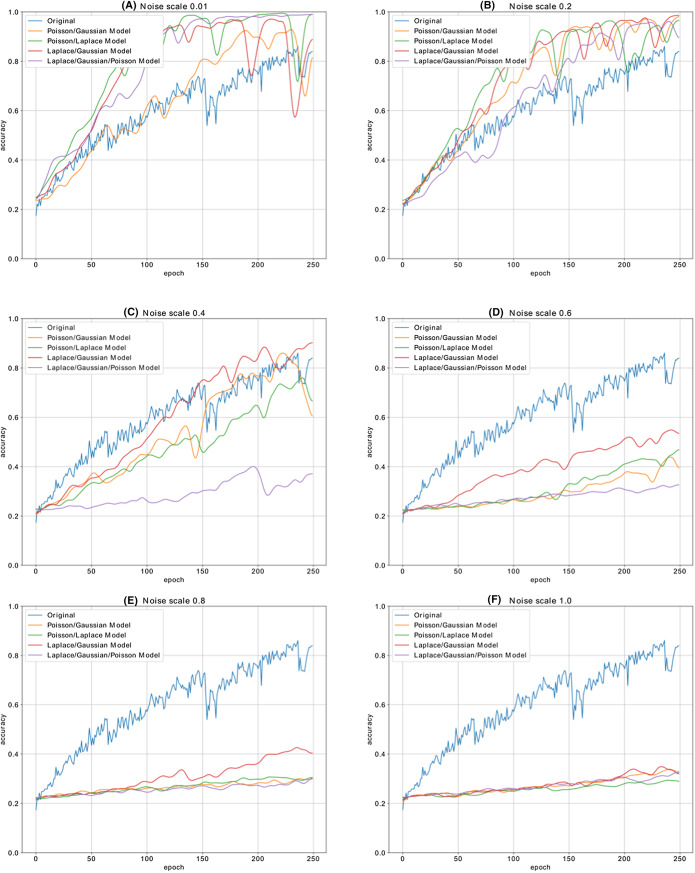
The different noise ratio accuracy results for the combination of additive Laplace/Gaussian/Poisson and original CNN model’s accuracy results. Noise scale: (A) 0.01; (B) 0.2; (C) 0.4; (D) 0.6; (E) 0.8 and (F) 1.0.

Figure 13 shows the accuracy change in each iteration of the CNN model, which is trained with the malware dataset containing a different amount of noise with different combination of noise models. The performance results of five CNN models, whose dataset is enriched by using combination of Additive Laplace, Additive Gaussian and Additive Poisson methods, are better than the CNN model’s classification performance that is trained only with the original training data set. When the noise ratio is 0.4, the original CNN model’s classification result is better than the CNN model with the several combination of noise injection methods.

Table 4 shows the accuracy changes with different noise methods and different noise ratio. The fields shown as bold on the table show the best accuracy value of the column. The best accuracy value for Poisson noise is obtained with 0.902 and 0.3 noise ratio, the best accuracy value for Gaussian noise is obtained with 0.922 and 0.4 noise ratio, and the best accuracy value for Laplace noise is obtained with 0.819 and 0.2 noise ratio. According to the table, we obtain the best classification performance with the Gaussian noise’s 0.4 noise ratio.

**Table 4:** Noise injection accuracy results. The bold entries show the best values.

Noise ratio	Orginal model	Poission	Gaussian	Laplace
0.01	0.83	**1.00**	0.96	**0.99**
0.2	0.83	0.95	**0.99**	0.87
0.4	0.83	0.95	0.95	0.98
0.6	0.83	0.60	0.98	0.92
0.8	0.83	0.49	0.94	0.35
0.0	0.83	0.33	0.80	0.48

Table 5 shows the accuracy changes with the different combination of noise methods and different noise ratio. The fields shown as bold on the table show the best accuracy value of the column. The best accuracy value for Poisson/gaussian noise is obtained with 0.93 and 0.2 noise ratio, the best accuracy value for Poisson/laplace noise is obtained with 0.95 and 0.01 noise ratio, the best accuracy value for Laplace/gaussian noise is obtained with 1.00 and 0.01 noise ratio.

**Table 5:** The best accuracy rates for the combination of each noise type. The bold entries show the best accuracy values.

Noise	Org	Poisson/Gaussian	Poisson/Laplace	Laplace/Gaussian	All
0.01	0.83	0.90	**0.95**	**0.98**	**0.96**
0.2	0.83	**0.93**	0.90	0.95	0.95
0.4	0.83	0.90	0.71	0.90	0.42
0.6	0.83	0.47	0.52	0.38	0.76
0.8	0.83	0.52	0.47	0.76	0.66
0.0	0.83	0.76	0.52	0.47	0.76

**Figure 14:**
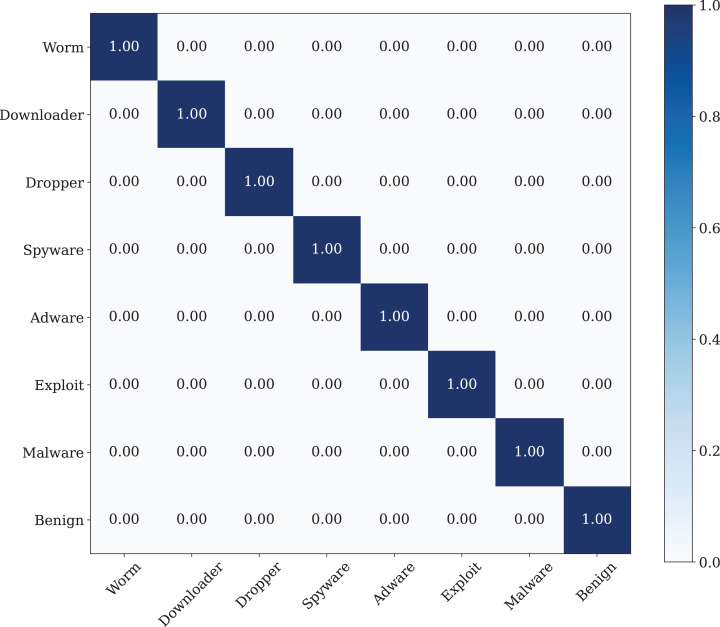
The confusion matrix of the CNN model with best data noise injection ratio.

The best classification performance is performed by using the Poisson noise with 0.01 value has a 100% classification performance. Figure 14 shows the confusion matrix of the malware detection model with the best classification performance.

## Conclusion and future work

The primary purpose of this research study is to detect malware families in a metamorphic malware environment using an image augmentation enhanced deep CNN model. The architecture of the model consists of three main components: image generation from malware samples, image augmentation, and classifying the malware families by using CNN models. In the first component, the collected malware samples are converted into binary representation using the windowing technique. The imgaug Python library is used to apply image augmentation techniques in the second component. The dataset is enhanced using additive noise techniques such as Gaussian, Laplacian, and Poisson. We apply it to our dataset with different hyper-parameters to demonstrate the proposed model’s effectiveness and classification performance. Finally, we train our classifier on our public dataset with seven different classes, including Worm, Downloader, Spyware, Adware, Exploit, Malware and 346 Benign. The model reaches its steady-state after the 80th epoch.

We observe that the training dataset shows more stable progress as compared to the test dataset, although both progress together. We apply four different metrics to evaluate the classification accuracy, such as the overall prediction accuracy, average recall, average precision and *F*_1_-score. The confusion matrix results indicate that the classification model performance is not good enough for malware detection. The classification performance of the model obtained with conventional CNN is relatively low. According to these results, a standard CNN model with an RGB type 3-channel image training dataset is not suitable for malware detection and classification. The augmentation is measured with varying noise ratio to assess the effectiveness of the learning algorithm. This article’s main contribution is to propose a data augmentation enhanced malware family classification model that exploits augmentation for variants of malware clones and takes advantage of CNN to improve image classification. It is evident from the results of this research that the data augmentation based on 3-channel image classification can significantly influence the performance of malware family classification. In future work, we intend to classify the correctly labeled dataset using the malware images method. We also plan to apply other sequential data classification algorithms used before deep learning.

## Additional Information and Declarations

### Data Availability

The following information was supplied regarding data availability: Data is available at GitHub: https://github.com/ocatak/malware_api_class
